# Transnationalism and care of migrant families during pregnancy, postpartum and early-childhood: an integrative review

**DOI:** 10.1186/s12913-020-05632-5

**Published:** 2020-08-24

**Authors:** Lisa Merry, Sarah Fredsted Villadsen, Veronik Sicard, Naomie Lewis-Hibbert

**Affiliations:** 1 Faculty of Nursing, University of Montreal, Montreal, Canada; 2 Department of Public Health, University of Copenhagen, Copenhagen, Denmark; 3 School of Kinesiology and Exercise Science, University of Montreal, Montreal, Canada; 4 School of Public Health, University of Montreal, Montreal, Canada

**Keywords:** Transnationalism, Migration, Pregnancy, Maternity care, Early childhood, Health and social care, Parenthood

## Abstract

**Background:**

Migrant families’ transnational ties (i.e., connections to their countries of origin) may contribute to their hardships and/or may be a source of resiliency. A care approach that addresses these transnational ties may foster a positive identity and give coherence to experiences. We conducted an integrative review to determine what is known about transnational ties and the care of migrant families during pregnancy, postpartum and early childhood.

**Methods:**

We searched 15 databases to identify literature reporting on a health or social program, service, or care experience of migrant families during pregnancy up to age five in a Western country (i.e., Canada, US, Australia, New Zealand or a European country). Information regarding if and how the service/program/care considered transnational ties, and care-providers’ perceptions of transnational ties, was extracted, analyzed and synthesized according to transnational ‘ways of belonging’ and ‘ways of being’.

**Results:**

Over 34,000 records were screened; 69 articles were included. Care, programs and services examined included prenatal interventions (a mhealth app, courses, videos, and specialized antenatal care), doula support, maternity care, support groups, primary healthcare and psycho-social early intervention and early childhood programs. The results show that transnational ties in terms of ‘ways of belonging’ (cultural, religious and linguistic identity) are acknowledged and addressed in care, although important gaps remain. Regarding ‘ways of being’, including emotional, social, and economic ties with children and other family members, receipt of advice and support from family, and use of health services abroad, there is very little evidence that these are acknowledged and addressed by care-providers. Perceptions of ‘ways of belonging’ appear to be mixed, with some care-providers being open to and willing to adapt care to accommodate religious, cultural and linguistic differences, while others are not. How care-providers perceive the social, emotional and economic ties and/or the use of services back home, remains relatively unknown.

**Conclusion:**

Significant knowledge gaps remain regarding care-providers’ perceptions of transnational ‘ways of being’ and whether and how they take them into account, which may affect their relationships with migrant families and/or the effectiveness of their interventions. Continued efforts are needed to ensure care is culturally safe for migrants.

## Background

Having and raising children can be challenging, particularly during resettlement in a new country [1]. Migrant parents (e.g., immigrants, refugees) may struggle financially and encounter difficulties managing cultural differences related to childbearing and parenting, for example different approaches for disciplining and socializing children. Many families must also adjust to shifting family relationships associated with changes in the family structure and gender dynamics [2–5] and cope with the loss of social networks, discrimination and navigating new health and social systems [2, 3, 6–10]. Migrant families stay connected to their countries of origin and their transnational ties may contribute to their hardships and/or may be a source of strength and resiliency [1].

Transnational ties include ‘ways of being’ as well as ‘ways of belonging’ [11, 12]. ‘Ways of being’ refers to the transnational practices and relations that are sustained (i.e., economic, social, emotional and/or civic relationships and involvement) with the home country whereas ‘ways of belonging’ refers to the maintenance of a transnational identity (i.e., religious, cultural/ethnic, linguistic, and/or political) [11, 12]. Transnational ‘ways of being’ and ‘ways of belonging’ can influence the childbearing and parenthood experiences in a new country in a multitude of ways. Many migrant families continue to support family via sending remittances and this may affect them financially [2, 13–16]. A number of families also parent children and care for elderly family members from a distance and this too may cause financial strain as well as psychological distress [3, 15, 17–19]. Staying connected to family and friends back home may enhance feelings of isolation and loneliness and hinder cultural adaptation, while use of health services back home may have an effect on continuity of care [20–22]. Conversely, transnational ties can serve as a resource for migrant families. Connecting and visiting the home country can help maintain language and to pass on traditions to children and serve as a source of support for parenting [15, 16, 23–26]. Bilingualism may contribute to social and economic capital; maintaining culture can promote family closeness and well-being and help resist oppression [5, 7, 24, 27]. Migrants may rely on advice from family members for staying healthy during pregnancy and postpartum, for parenting (e.g., nutrition, discipline, socialization) and for resolving family conflicts [24, 25]. Family and friends back home may also provide emotional support and childcare either for children who remained behind, or for infants/children who are sent to the home country temporarily [15, 16, 28–31]. Health services may also be accessed for reasons of cost or cultural acceptability, and work/business activities may be an additional source of income [21, 32–34]. Continued engagement civically and politically may also function as a coping strategy to deal with cultural and social losses associated with migration [7, 30].

In the health field there is a growing interest in transnationalism and its relevance to health and care [35, 36]. In mental health it has also been shown that an approach in care that acknowledges and addresses transnational ties may foster a positive identity and give meaning and coherence to migrant families’ experiences [37, 38]. The early years (0–5 years) is a time when migrant families may first come into contact with health and social services, especially around the time when a child is born. It’s also an opportune moment for intervention, since pregnancy and early childhood are crucial periods for child development and it is also when parents may feel overwhelmed. During this period families may interact with a range of care-providers (e.g., nurses, doctors, social-workers, educators) in various settings such as hospitals, clinics/health centers, early childhood programs and community organizations. Some may be involved in specialized programs which aim specifically to support more vulnerable families, particularly in the context of adapting to a new culture and language [31]. General care as well as specialized programs may offer services to enhance parenting skills, foster child development, promote a healthy lifestyle, and provide social connection for families experiencing isolation [31, 39–42]. In this literature review, we sought to determine what is known regarding transnational ties and the health and social care of migrant parents/families during pregnancy, postpartum and early childhood in Western, high-income countries. The following research questions were addressed: 1- Do health and social services aimed towards promoting health and providing support to parents/families during pregnancy, postpartum and early childhood (0–5 years) consider the transnational contexts and experiences of migrants? 2- How do care-providers address transnational ties in their care and interventions with migrant families during pregnancy, postpartum and early-childhood? and 3-What are these care-providers’ perspectives on migrant families’ transnational ties to their home country?

## Methods

We conducted an integrative review [43]. This type of review allows for the inclusion of diverse literature (research and discussion papers). We searched broadly to identify papers that described either the *care-giving* experiences of health or social care professionals or the *care-receiving* experiences of migrant families during pregnancy, postpartum or early childhood in Western countries. We also included papers that generally described or reported on the development or evaluation of a health or social program/intervention, or service for migrant families during pregnancy up to age five. All papers were then assessed to determine if there is any evidence that health and social services supporting migrant families during pregnancy, postpartum and early childhood consider and address transnational ties, including ‘ways of being’ and ‘ways of belonging’, during care with these families. We also assessed to see what information exists on care-providers’ perspectives of transnational ties.

### Search strategy

Searches were conducted by VS and NLH in 15 online databases: EMBASE, Web of Science, Medline, PubMed, PsycINFO, CINAHL, Scopus, Family Studies Abstracts, Global Health, Social Sciences Abstracts, Social Work Abstracts, Campbell Collaboration, Social Services Abstracts, Sociological Abstracts, and Dissertations and Thesis. The searches in CINAHL and EMBASE excluded Medline records. We selected the databases and developed the search strategy in consultation with a university librarian. Subject headings/descriptors and keywords were identified to capture key concepts including migration (e.g., immigrant, refugee, transient, asylum, resettlement), parenthood (e.g., mother, father, child-caregiver), early childhood (e.g., child, toddler, infant, pre-schooler, newborn, early childhood, child birth, pregnancy), and health/social services (e.g., health services, health care services, social services, counseling, mental health, social support). Terms were adjusted according to the vocabulary used in each database. The searches for parenthood, early childhood, migration, and health/social services were then combined using the AND Boolean operator. Keywords were searched within titles, abstracts, and keywords of articles. To ensure the literature obtained was relevant to the current migration and health and social care context, we limited the searches to the last 15 years (January 2004 to December 2018; from January to July 2019 we set-up alerts to notify us when potentially relevant literature was published after the initial searches were conducted). We also applied a language restriction (English and French) based on our language capacities. An example of a detailed database search strategy (Web of Science) is shown in Table 1.

**Table 1:** Web of Science database search strategy

1	mother* OR father* OR parent* OR grandparent* OR grandmother* OR grandfather* OR famil* OR sibling* OR household* OR “in-law*” OR caregiver* OR (child NEAR/3 caregiver*) OR “child-caregiver” OR dad* OR mom* OR pregnan* OR childrearing OR “child rearing”
2	child* OR infant* OR newborn* OR baby OR babies OR “pre-schooler*” OR pregnan* OR offspring* OR toddler OR kid
3	Immigra* OR migra* OR emigra* OR refugee* OR asylum OR transient* OR undocumented OR resettl* OR settl* OR transmigration OR reestablish* OR relocate* OR exodus OR expatri* OR displac* OR exile OR deserter* OR deport* OR transnational
4	((Social OR support) NEAR/4 service*) OR ((social OR emotional OR psycho*) NEAR/4 support) OR counsel$ing OR nurs* OR intervention* OR “wellness program” OR “support services” OR therap* OR emergenc* OR ((intensive OR critical OR primary OR mental) NEAR/4 care) OR preventi* OR health* OR clinic* OR psycho* OR “caregiver NEAR/3 support”
5	1 and 2 and 3 and 4
6	Limit 5 to January 2004-Current
7	Limit 6 to English or French

In addition to the database searches we also hand-searched the reference lists of included papers. The same time and language limitations were applied.

### Inclusion and exclusion criteria

Table 2 provides a summary of the inclusion and exclusion criteria. Peer-reviewed publications including research (qualitative, quantitative, or mixed methods) and discussion papers, were considered. We also considered dissertations, however if results were published in an article, we only kept the latter. Literature reviews, study protocols, commentaries and books were excluded. Literature was included if it reported on a health or social program, intervention, or service, or if it described the care-giving experiences of health or social care professionals or if it examined the care-receiving experiences of families during pregnancy, postpartum or early childhood in Canada, the US, Australia, New Zealand or European countries. The care/service/program/intervention had to involve general health promotion activities; to keep the results manageable, literature focusing on care/services/programs/interventions that targeted only one aspect of health (e.g., breastfeeding, nutrition, dental care) were excluded. Papers must have included and examined or discussed migrant parents’ (mothers, fathers, and extended family involved in parenting) care experiences and/or described or discussed implications for the care of migrant parents/child caregivers (i.e., if the focus was only on children’s experiences or outcomes, the paper was excluded). Literature that examined care and services targeting families with parents or children who were ill or who had disabilities, or that focused on childhood without specifying child ages, or that did not report results for, or discuss pregnancy, postpartum or early childhood specifically, were excluded. “Migrant” was defined as anyone born outside of the country (including migrants without status); migration could have been for any reason (e.g., forced, economic) and could have been temporary or permanent [44]. If both migrants and non-migrants were included in a study or discussion paper, we retained the article only if migrant parents’ experiences and/or implications for care of migrant parents/child-caregivers were reported and discussed separately from those of non-migrants.

**Table 2:** Inclusion and exclusion criteria

Inclusion Criteria	
1. Peer reviewed publications and dissertations	
2. Reported on a health or social program/intervention or service, or described the care-giving or care-receiving experiences during pregnancy, postpartum and/or early childhood	
3. Examined or discussed migrant parents’ (mothers, fathers, and extended family) care experiences and/or implications for the care of migrant parents/child caregivers	
4. The receiving country was Canada, United States, Australia or a European country	
Exclusion Criteria	
1. Literature reviews, study protocols, commentaries and books	
2. Dissertations if published in articles (only the published articles were retained)	
3. The care/service/program/intervention targeted only one aspect of health (e.g., specialized programs for breastfeeding, nutrition, or dental care) or was not aimed at promoting health and/or it focused on families with parents or children who were ill or who had disabilities	
4. The child ages were not specified or results or discussion points on early childhood were not reported/discussed separately from those of other child age groups	
5. The focus was second or other generation migrants or mixed families (family members with migrant and non-migrant statuses)	
6. The focus was medical tourists (i.e., those who came to the receiving country only for care)	
7. Migrant parents’ experiences/implications for care of migrant parents/child caregivers were not reported or discussed separately from non-migrants	
8. Reported or described health or parenthood outcomes or experiences without any mention of health or social care experiences/interactions or implications for care of migrant parents	

All citations were downloaded and managed using Mendeley Desktop software (version 1.19.4). VS and NLH conducted the screening of all titles and abstracts to select potentially eligible literature. Full papers were then retrieved and reviewed to confirm eligibility. If eligibility was uncertain, a decision was made via group discussion. LM reviewed and confirmed the final selection of the included literature.

### Data extraction, analysis and synthesis

For all eligible literature, we extracted and entered data into an Excel database. Data extracted included basic descriptors: the year, language and type of publication (original research, discussion paper, dissertation); the location of the study/discussion (Canada, US, Australia, New Zealand and/or European countries), information about the type of service/program/intervention and/or care given/received, the objective of the paper, and if applicable, the research design, methods, and population studied (parents and/or care-providers). For studies that examined parents, we noted who was included (mothers and/or fathers), their migration status, countries/regions of origin, length of time in the country, and/or their ethnicity. To address the research questions, information regarding if and how the service/program/care/intervention addressed transnationalism (ways of being and/or ways of belonging), as well care-providers’ perspectives on transnational ties, were also extracted. We also noted any general reference made to transnational ties in the article even if it was not in relation to the intervention/service/program/care. LM, with the support of a research assistant, assessed the methodological quality of empirical studies (qualitative, quantitative and mixed methods) using the Mixed Methods Appraisal Tool (MMAT) [45]. The MMAT uses a checklist (Yes/No/can’t tell) format with five questions that vary depending on the type of research being assessed. For qualitative research, studies were appraised on the appropriateness of the methodology and data collection methods, the adequacy of the data and its interpretation and the coherence between the data collection, analyses and findings. Quantitative studies were evaluated on the appropriateness of the sampling, randomization (if relevant), measurement and analyses, and the risk for bias. Mixed methods studies were assessed for the suitability of using a mixed methods design for the research questions being addressed, the methodological quality of each component, and the appropriateness of the ‘mixing’ of the qualitative and quantitative aspects of the study. No studies were excluded based on the quality assessment. All information gathered from the literature was synthesized into summary tables and text.

## Results

**Fig. 1:**
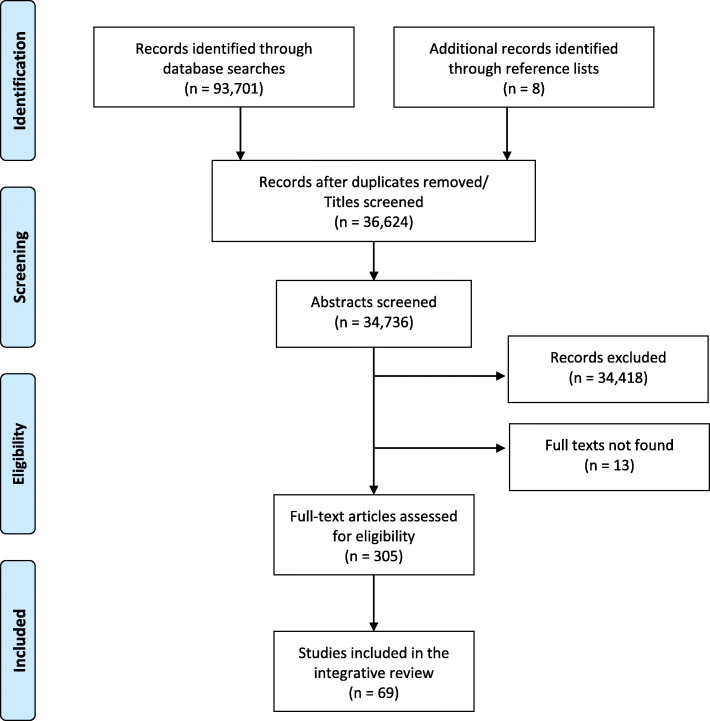
PRISMA Flow Diagram

The database searches resulted in over 93,000 records (see Additional file 1 for the search results for each database). Once duplicates were removed and the initial screening was conducted, 318 records were considered for inclusion. Thirteen articles could not be located and 236 were excluded following reading of the full text. The main reasons for exclusion at this stage were: 1- the child ages were not specified and/or results or discussion points were reported for all child age groups together; 2- migrant parents’ maternity and parenthood experiences were described generally without any reference to health or social care experiences/interactions or implications for care; and 3- results and implications for migrants and non-migrants were not reported separately. Sixty-nine articles were included in the review; The PRISMA flow diagram is presented in Fig. 1 and a summary of the included literature is reported in Table 3.

**Table 3:** Description of included literature

#	1st Author year	Objective	Country Location	Care, program or intervention examined	Design/Methodology^a^	Sample & Data collection methods	Migrant groups included in the research^b^
1.	Akhavan 2012 [46]	To explore the experiences of doula support among foreign-born women in Sweden in the context of a “Community-Based Doula” intervention project	Sweden	Community-based doula	Qualitative descriptive	10 mothers	Tunisia, Somalia, Yemen, Iraq, Turkey, Morocco, Azerbaijan
						Interviews	
							LOT^*^: 1 to 3.5 years
							Refugee, family sponsored
2.	Akhavan 2012 [47]	To describe and analyze midwives’ experiences of doula support for immigrant women in Sweden	Sweden	Community-based doula	Qualitative descriptive	10 midwives	N/A
						Interviews	
3.	AlJaberi 2018 [48]	To develop a comprehensive understanding of the pregnancy health and social needs of low-income Caribbean immigrants towards informing the development of a mHealth intervention	United States	Pregnancy mobile health intervention	Qualitative descriptive	12 mothers	Caribbean
						Focus groups	Immigrant
4.	AlJaberi 2018 [49]	To understand the emotional, physical, information and social challenges affecting low-income women’s prenatal well-being practices towards developing a mHealth intervention for these women	United States	Pregnancy mobile health intervention	Qualitative descriptive	12 mothers	Caribbean
						Focus groups	Immigrant
5.	Almeida 2014 [50]	To verify whether there are differences regarding women’s perceptions of quality and appropriateness of care received between immigrant and native women during pregnancy and postpartum	Portugal	Healthcare during pregnancy and postpartum	Qualitative descriptive	31 mothers	African countries (Portuguese-speaking), Brazil, Eastern European countries
						Interviews	
							LOT: 2 to 20 years
							Immigrant, undocumented
6.	Almeida 2014 [51]	To provide qualitative information on the access, use and perceived quality of care during pregnancy and early motherhood, reported by a group of immigrants in a large urban area in northern Portugal; Portuguese women were also interviewed for comparison	Portugal	Care during pregnancy and early motherhood	Qualitative descriptive	31 mothers	African countries (Portuguese-speaking), Brazil, Eastern European countries
						Interviews	
							LOT: 2 to 20 years
							Immigrant, undocumented
7.	Anderson 2014 [52]	To explore recent immigrant mothers’ experiences accessing and utilizing primary healthcare for their young children 1–5 years old	Canada	Primary healthcare for children 1 to 5 years old	Qualitative descriptive	32 mothers	Latin American, Sri Lankan Tamil
						Interviews	LOT: ≤ 5 years
							Arrived as refugee claimant or was family-sponsored
8.	Ayers 2018 [53]	To explore maternal health care provider’s perspective of barriers in providing care to Marshallese women and providers perceived barriers of access to care among Marshallese women	United States	Maternal healthcare	Phenomenology	15 nurses, 2 obstetricians and 2 other healthcare providers (unspecified)	N/A
						Focus groups and interviews	
9.	Aubé 2019 [31]	To describe the challenges and protective factors that affect the well-being of migrant mothers and how La Maison Bleue, a community-based perinatal health and social centre, strengthens resilience among these families	Canada	A community-based perinatal health and social centre	Focused ethnography	24 mothers	Bangladesh, Saint-Lucia, Democratic Republic of Congo, Morocco, Mexico, Cameroon, Eritrea, Pakistan, Sri Lanka, India, Algeria
						Interviews and observations	
							LOT: < 1 to 11 years
							Immigrant, refugee, asylum seeker, undocumented
10.	Balaam 2016 [54]	To explore the experience of voluntary and non-statutory sector workers supporting asylum-seeking and refugee women during pregnancy and early motherhood	United Kingdom	Voluntary and non-statutory support for childbearing refugee and asylum seeking women	Qualitative descriptive	19 volunteer and non-statutory (paid and unpaid) workers	N/A
						Individual and focus group interviews	
11.	Barkensjo 2018 [55]	To describe women’s experiences of clinical encounters throughout pregnancy and childbirth, when living as undocumented migrants in Sweden	Sweden	Maternity care	Qualitative descriptive	13 mothers	Macedonia, Romania, Bosnia, Albania, Somalia, Afghanistan, Serbia, Chechnya, Morocco, Kosovo
						Interviews	
							Undocumented, EU citizens without residency permits
12.	Barona-Vilar 2013 [56]	To explore the experiences and perceptions of parenthood and maternal health care among Latin American women living in Spain	Spain	Maternal health care	Qualitative descriptive	26 mothers and 24 midwives	Bolivia, Ecuador
						Focus groups	LOT: 1 to 9 years
							Immigrant, undocumented
13.	Beaudet 2016 [22]	To evaluate a support-group intervention developed collaboratively between a community organization and a local health clinic to address isolation and support recently-arrived immigrant mothers with children aged 0 to 2 years	Canada	Community support-group intervention for immigrant mothers	Qualitative descriptive	13 mothers, 2 administrators, 2 support group workers & 1 social worker (support group committee), Mothers and children participating in the support group, and Administrators & workers from the community organization	China, Colombia, Korea, Egypt, France, Honduras, India, Iran, Iraq, Japan, Lebanon, Libya, Poland, Singapore, Taiwan
							LOT: 8 women < 2 years, others longer
							Immigrant
						Interviews, discussion groups, observations of support group sessions and committee meetings, and a review of documents (support-group participation logbook, child assessments, support-group journal)	
14.	Bircher 2009 [57]	To describe the challenges of migrant farm workers during pregnancy and to suggest ways that advanced practice nurses can provide cost effective, competent professional care to reduce or eliminate the obstacles to prenatal care for this population	United States	Prenatal care offered by a nurse practitioner	Discussion paper		
15.	Boerleider 2014 [58]	To gain insight into how Dutch postnatal care providers - maternity care assistants -address issues encountered when providing care for non-western women	Netherlands	Postnatal care	Qualitative descriptive	15 maternity-care assistants	N/A
						Interviews	
16.	Briscoe 2009 [59]	To explore the experience of maternity care by asylum seekers and one refugee	United Kingdom	Maternity care	Multiple case study	4 mothers	Afghanistan, Congo, Rwanda, Somalia
						Photographs, observations, and	LOT: < 1 year to just under 3 years
						interviews	Refugee, asylum seeker
17.	Busch 2018 [60]	To investigate challenges and possible solutions in a specialized early childhood education and care (ECEC) program for refugee children	Germany	A specialized ECEC for refugee children	Mixed-methods (qualitative descriptive followed by a survey based on the qualitative data)	28 early-childhood educators	N/A
						96 early-childhood educators (a second sample)	
						Interviews and questionnaire	
18.	Carolan 2010 [61]	To explore the experiences and concerns of an African-born sample of pregnant women receiving antenatal care in Melbourne	Australia	Antenatal care	Qualitative descriptive	18 mothers	Ethiopia, Sudan, Eritrea, Kenya, Somalia
						Interviews	LOT: < 1 year to 2 years
							Refugee, family-reunification visa, immigrant
							Dinka, Amharic, Christian
19.	Clark 2007 [62]	To identify Mexican American mother’s expectations from children’s health care services (during the first 19 months of their child’s life)	United States	Children’s health care	Focused ethnography	28 mothers	Mexico
						Interviews	Immigrant, undocumented
20.	Coley 2012 [41]	To 1) describe the development of the Moms Matter support group; 2) illustrate the effects of incorporating cultural competence and social support in childbirth education; and 3) suggest implications for the future development of pregnancy support programs for diverse immigrant populations	United States	Support group to enhance prenatal and postnatal education for immigrant mothers	Qualitative descriptive	7 mothers	Nigeria, Ghana, Nepal, Mexico, India, Jordan
						Interviews	
							Immigrant
21.	Coutinho 2014 [63]	To identify the unmet expectations of Portuguese immigrant women, for the National Health System, during pregnancy, childbirth and postpartum	Portugal	Maternity care	Qualitative descriptive	82 mothers	Brazil, Ukraine, China, Moldova, Russia, France
						Interviews	
							Immigrant
22.	Doering 2015 [64]	To explore how some Japanese women experienced pregnancy, labor and birth care in New Zealand	New Zealand	Maternity care	Qualitative descriptive	13 mothers	Japan
						Interviews and a focus group	LOT: 2 to 19 years
							Immigrant
23.	Degni 2012 [65]	To explore physicians and nurses/midwives’ communication when providing reproductive and maternity health care to Somali women in Finland	Finland	Maternity care	Qualitative description	10 obstetricians, 7 nurses, and 8 midwives	N/A
						Individual and focus group interviews	
24.	Degni 2014 [66]	To explore immigrant Somali women’s experiences of reproductive and maternity health care services and their perceptions of the service providers	Finland	Maternity care	Qualitative descriptive	70 mothers	Somalia
						Focus groups	LOT: ‘recently migrated’
							Refugee
25.	Dempsey 2016 [67]	To explore migrant Eastern European women’s experience of pregnancy in Ireland	Ireland	Maternity care	Grounded theory approach	12 mothers	Poland, Lithuania, Hungary, Czech Republic
						Interviews	LOT: 1 to 8 years
							Economic immigrant
26.	DeStephano 2010 [68]	To determine the acceptability of a culturally tailored prenatal health education video series for Somali women and explore health providers’ perceptions regarding usefulness of the videos in facilitating improved client–provider communication	United States	Culturally tailored prenatal health education video series for Somali women	Quantitative descriptive with a qualitative component	22 mothers, 2 fathers and obstetricians who cared for the 22 women	Somalia
							Refugee
						Questionnaires	
27.	Gabai 2013 [69]	To explore the experiences of patients and maternity care-givers in a maternity context	France	Maternity care	Grounded theory	4 mothers, 10 obstetricians and midwives	Lebanon, Ivory Coast, Democratic Republic of Congo, Tunisia
						Interviews	LOT: 3 to 11 years
							Immigrant
28.	Grewal 2008 [70]	To describe new immigrant Punjabi women’s perinatal experiences and the ways that traditional beliefs and practices are legitimized and incorporated into the Canadian health care context	Canada	Perinatal care	Qualitative descriptive	15 mothers, 5 public health nurses	India
						Interviews and a focus group	LOT: ≤ 5 years
							Immigrant
							Punjabi
29.	Higginbottom 2013 [71]	To map out the experiences of immigrant Sudanese women in maternity services	Canada	Maternity services	Focused ethnography	12 mothers	Sudan
						Focus group interviews	LOT: ≤ 5 years
							Refugee
30.	Hill 2012 [72]	To describe Somali immigrant women’s health care experiences and beliefs regarding pregnancy and birth	United States	Maternity care	Qualitative descriptive	18 mothers	Somalia
						Focus group interviews	LOT: 1.5 to 12 years
							Refugee
31.	Hurley 2014 [73]	To investigate the challenges and innovative practices in early childhood special education (ECSE) services for preschool aged children who are refugees	United States	Early childhood special education (ECSE) services	Qualitative descriptive	28 early-childhood educators Interviews	N/A
32.	Iliadi 2008 [74]	To examine whether refugee women resettled in Greece, receive antenatal care and to explore possible factors that may influence their attitude towards maternal care	Greece	Maternity care	Focused ethnography	26 mothers Interviews	Iraq, Iran, Sudan, Lebanon, Syria, Afghanistan, Armenia, Turkey, Albania, Serbia, Zaire Refugee
33.	Karl-Trummer 2006 [75]	To evaluate a prenatal training course developed for pregnant migrant/ethnic women	Italy	Prenatal training course for migrant women	Mixed-methods (qualitative descriptive in conjunction with a survey)	41 mothers and 32 healthcare providers	Turkey, India, Pakistan
			Austria				Immigrant
						Interviews and questionnaire	
34.	Lebiger-Vogel 2019 [76]	To present and discuss the FIRST STEPS project in Belgium and the FIRST STEPS project in Frankfurt and Berlin (FIRST STEPS is a prevention/support intervention offered to immigrant women in early childhood and which aims to optimize the early developmental environment of children)	Belgium	Early-childhood support program for immigrant parents	Discussion paper		
			Germany				
35.	Lyberg 2012 [77]	To illuminate midwives’ and public health nurses’ perceptions of managing and supporting prenatal and postnatal migrant women in Norway	Norway	Maternity care	Qualitative descriptive	5 midwives and 1 public health nurse	N/A
						Focus groups	
36.	Lyons 2008 [78]	To explore the experiences, understanding and perspectives of maternity service providers when working with ethnic minority women in Dublin maternity services during 2002 and 2003	Ireland	Maternity services	Grounded theory approach	42 obstetricians, midwives, nurses, and key informants from specialized areas of infection control, social services and bereavement services	N/A
						Focus groups and interviews	
37.	Mangrio 2017 [79]	To shed light on the experience of non-European immigrants with Sweden’s child health care system	Sweden	Child health care	Qualitative descriptive	14 mothers and 5 fathers	Afghanistan, Chile, India, Iraq, Kurdistan, Kuwait, Lebanon, Pakistan, Palestine, Venezuela, Vietnam
						Interviews	
							LOT: 2 to 22 years
							Immigrant, refugee
38.	McLaughlin 2012 [80]	To explore the lived experiences of parenting amongst a group of Burmese refugee mothers and their perceptions of how facilitated playgroups assist them in their parenting role	Australia	Facilitated playgroup	Phenomenology	9 mothers, 2 playgroup staff and 1 kindergarten teacher Focus groups, interviews	Burma Refugee
39.	Merry 2011 [81]	To gain a greater understanding of the barriers asylum seeking women face in accessing health and social services postpartum	Canada	Health and social services postpartum	Qualitative descriptive	112 mothers	Africa, Asia, Europe, Latin America
						Review of nurses’ notes	LOT: ≤ 5 years
							Asylum seeker
40.	Mukasa 2016 [82]	To 1) understand the disparities in access to maternal and child health (MCH) services experienced by recent African immigrant mothers in the United States; 2) explore circumstances that led to MCH access disparities experienced by this population; and 3) understand how access disparities affected participants’ overall experience of seeking MCH care services	United States	Maternal and child health services	Phenomenology	11 mothers	Sub-Saharan Africa
						Interviews	LOT: 1.5 to 4 years
							Immigrant, refugee, asylum seeker
41.	Nabb 2006 [83]	To explore the perceptions of pregnant asylum-seekers in relation to the provision of maternity care while in emergency accommodation in the UK	United Kingdom	Maternity care	Qualitative descriptive	10 mothers and 5 healthcare professionals	Africa, Asia, Eastern Europe
							Asylum seeker
						Interviews	
42.	Ng 2011 [84]	To understand the difficulties health care professionals face when delivering prenatal care to immigrant women	Canada	Prenatal care	Qualitative descriptive	3 midwives, 5 nurses practitioners, and 2 obstetricians	N/A
						Interviews	
43.	Ny 2006 [85]	To describe how men from the Middle East experience Swedish maternity and child health care	Sweden	Maternal and child healthcare	Qualitative descriptive	16 fathers	Middle-East
						Interviews and focus groups	LOT: 1–15 years
							Immigrant
44.	Owens 2016 [86]	To explore the perceptions of care experienced by refugees and migrant women of culturally and linguistically diverse backgrounds who had participated in a community-based antenatal service specializing in maternity care for multicultural women	Australia	Community-based antenatal service specializing in maternity care of women from culturally and linguistically diverse backgrounds	Phenomenology	I2 mothers	Indonesia, Pakistan Vietnam, Iran, Sudan, Burma, Thailand
						Interviews	
							LOT: 1–10 years
							Immigrant, refugee
							Bhatak, Baloch, Catholic, Muslim, Bari, Chin, Karen
45.	Pelaez 2017 [87]	To explore health care professionals’ perspectives of challenges newly-arrived migrant women to Canada coming from non-western countries face when needing maternity care in order to better understand clinical practices towards these women	Canada	Maternity care	Multiple case study	3 family physicians, 5 obstetricians, 4 medical residents, 1 nutritionist, 1 anesthesiologist, 7 social workers, 1 art therapist, 1 psychologist, 1 spiritual consultant, and 39 nurses	N/A
						Interviews	
46.	Phillimore 2016 [88]	To examine the reasons why migrants’ access to antenatal care is poor	United Kingdom	Antenatal care	Mixed-methods (Qualitative descriptive in conjunction with a questionnaire)	82 mothers and 18 community health staff, general practitioners, pregnancy outreach workers, hospital staff and third sector workers	28 countries including China, Iran, Pakistan, Poland, Zimbabwe
							LOT: ≤ 5 years
							Immigrant, refugee, asylum seeker, undocumented
						Questionnaire and interviews	
47.	Qureshi 2013 [21]	To describe the comparative birthing experiences of Pakistani immigrant women in Pakistan and the United States	United States	Maternity care	Ethnography	26 mothers	Pakistan
						Interviews	LOT: average of 12 years
							Immigrant
48.	Renzaho 2014 [89]	To explore the views and perceptions of migrant women in Dandenong, Australia, about sociocultural barriers and health needs during pregnancy and in the postnatal period	Australia	Pregnancy and postnatal care	Qualitative descriptive	35 mothers	Afghanistan, Africa, China, Palestine, Lebanon, Syria, Iran, Jordan
						Focus groups	
							LOT: 2 to 11 years
							Immigrant
49.	Rickmeyer 2015 [90]	To present preliminary results from a project that aims to evaluate the FIRST STEPS program, which is an early-childhood parenting support and child development intervention; preliminary results included attendance rates to the program, socio-demographics of the participating population and vignettes to illustrate some of the positive effects for families	Germany	Early-childhood support program for immigrant parents	Randomized control trial (with a qualitative component)	224 mothers	Ethiopia, Eritrea, Kenya, Sudan, Benin, Democratic Republic of Congo, Ghana, Nigeria, Mexico, Venezuela, Algeria, Egypt, Morocco, Tunisia, Bulgaria, Poland, Romania, Croatia, Kosovo, Montenegro, Serbia, Turkey, Afghanistan, India, Pakistan, Palestine, Saudi Arabia, Syria, China, Japan, Korea, Vietnam
						Questionnaires and vignettes	
							LOT: ≤ 3 years
							Immigrant
50.	Riggs 2012 [91]	To explore experiences of using maternal and child health services, from the perspective of families from refugee backgrounds and service providers	Australia	Maternal and child health services	Qualitative descriptive	87 mothers and 5 healthcare providers (nurses, other healthcare workers and bicultural workers)	Iraq, Burma, Lebanon, Bhutan, Sudan
							LOT: 1.5 to 8.5 years
							Refugee
							Karen, Assyrian Chaldean
						Focus groups and interviews	
51.	Riggs 2017 [92]	To describe the experiences of group pregnancy care for Karen women from Burma who have resettled in Melbourne, Australia	Australia	Group pregnancy care	Qualitative descriptive	19 mothers	Burma
						Focus groups	LOT: < 1 year to 10 years
							Refugee
							Karen
52.	Russo 2015 [93]	To explore the experiences of Afghan women living in Melbourne throughout pregnancy, birth, and early motherhood, and gain insight into the aspects of their experiences that they perceive as positively and negatively impacting their emotional wellbeing	Australia	Maternity care	Qualitative descriptive	38 mothers	Afghanistan
						Focus groups and interviews	LOT: 1 to 6 years
							Refugee
53.	Sanchez 2017 [94]	To describe Mexican immigrant women experiences of pregnancy and birth and to identify the approaches that midwives use when caring for these women	United States	Midwifery care	Qualitative descriptive	20 mothers and 5 nurse-midwives	Mexico
						Interviews	LOT: 16 were in US < 3 years, others longer
							Undocumented, immigrant
54.	Schmiedigen 2013 [95]	To describe the subjective experience of Brazilian women entering motherhood in the United States	United States	Maternity care	Interpretive phenomenology	8 mothers	Brazil
						Interviews	LOT: ≤ 10 years
							Immigrant
55.	Seo 2017 [96]	To understand Korean immigrant women’s common experiences and practices of utilizing health care services in the United States during childbirth	United States	Health care services during childbirth	Interpretive phenomenology	15 mothers	Korea
						Interviews	LOT: 1.5 years to 19 years
							Immigrant
56.	Shafiei 2012 [97]	To explore immigrant Afghan women’s views and experiences of maternity care in Melbourne, Australia	Australia	Maternity care	Mixed-methods design (survey followed by qualitative interviews)	40 mothers	Afghanistan
						Questionnaire and Interviews	LOT: half were ≤ 5 years, other half > 5 years
							Refugee
57.	Signorelli 2015 [98]	To describe the STARTTerS Early childhood program (a multimodal program that aims to support child development and trauma recovery, and enhance parenting confidence and skills), and to report the results from a community project with Karen and Mandaean refugee communities which aimed to better tailor services for these populations	Australia	Early childhood program for refugee families	Qualitative descriptive	48 male and female participants including parents, grandparents, other care-givers community leaders and other community members	Burma, Iraq
							LOT: very few were recent arrivals
							Refugee
							Karen, Mandaean
						Focus groups and interviews	
58.	Signorelli 2017 [99]	To explore the implementation of a model to address access and other challenges in early childhood work with refugee families and communities, with the intent to increase service uptake	Australia	Early childhood program for refugee families	Discussion paper		
59.	Stapleton 2013 [100]	To explore whether maternity care for women from refugee backgrounds attending a specialist antenatal clinic in a tertiary Australian public hospital, could be improved	Australia	Antenatal clinic for refugee women	Mixed-methods (retrospective cohort, survey, and qualitative descriptive)	4348 mothers (hospital data), 42 service-users (mothers), 147 hospital staff, 3 clinic staff, 3 hospital managers, 2 interpreting coordinators, and 5 key stakeholders	Africa and Middle East including Somalia, Sudan, Afghanistan, Burundi, Liberia Refugee
						Surveys, interviews, focus groups, hospital and clinic databases and chart audit	
60.	Stewart 2015 [101]	To evaluate a social support intervention (support groups consisting of like-ethnic and like-gender peers) for refugee new parents	Canada	Psychosocial intervention for refugee parents with young children (4 months to 5 years)	Mixed-methods (pre-test post-test design and qualitative descriptive)	38 mothers and 47 fathers 21 peer and professional mentors	Sudan, Zimbabwe
							LOT: < 5 years
						Interviews and questionnaires (social support, loneliness and isolation, coping, parenting stress)	Refugee, asylum seeker
61.	Stewart 2017 [102]	To examine support needs of African refugee new parents in Canada to guide development of a tailored support intervention	Canada	Psychosocial intervention for refugee parents with young children (4 months to 5 years)	Mixed-methods (qualitative descriptive and questionnaires)	29 fathers and 43 mothers (additional parents also participated in the group interviews but unclear how many), and 15 service-providers and 15 policy-influences/makers	Zimbabwe, Sudan
							LOT: < 5 years
							Refugee, asylum seeker
						Individual and group interviews and questionnaires (social support, coping)	
62.	Stewart 2018 [103]	To develop and test an accessible and culturally appropriate social support intervention designed to meet the support needs and preferences identified by African refugee parents of young children	Canada	Social support intervention for refugee parents with young children (4 months to 5 years)	Qualitative descriptive	47 fathers and 38 mothers	Sudan, Zimbabwe
						Individual and group interviews	LOT: < 5 years
							Refugee
63.	Tobin 2014 [104]	To explore midwives’ perceptions and experiences of providing care to women in the asylum process and to gain insight into how midwives can be equipped and supported to provide more effective care to this group in the future	Ireland	Maternity care	Qualitative descriptive	10 midwives	N/A
						Interviews	
64.	Vesely 2011 [105]	To gain greater understanding of the lived experiences of immigrant mothers of young children as they parented in the U.S. and interacted with the Early childhood care and education system	United States	Early childhood care and education mothers	Ethnographic and grounded theory approaches	41 mothers, 4 fathers	Ethiopia, El Salvador, Eritrea, Sudan, Ghana, Mexico, Ecuador, Egypt, Guatemala, Morocco, Somalia, Ghana, Argentina
						Interviews and observations	
							LOT: 2 to 21 years
							Refugee, undocumented, immigrant
65.	Wikberg 2012 [106]	To describe and interpret from an intercultural perspective, the perceptions and experiences of immigrant new mothers in maternity care in Finland	Finland	Maternity care	Focused ethnography	17 mothers	Australia, Bosnia, Burma, Colombia, Estonia, Hungary, India, Iraq, Russia, Thailand, Uganda, Vietnam
						Interviews, observations, and documents (information given to mothers, newspapers, websites and informal notes)	
							LOT: < 1 year to 10 years
							Refugee, immigrant, family sponsored, asylum seeker
							Kurdish
66.	Wikberg 2014 [107]	To illuminate immigrant new mothers’ experiences and perceptions of caring in maternity services in Finland	Finland	Maternity care	Focused ethnography	17 mothers	Australia, Bosnia, Burma, Colombia, Estonia, Hungary, India, Iraq, Russia, Thailand, Uganda, Vietnam
						Interviews, observations, and documents (information given to mothers, newspapers, websites and informal notes)	
							LOT: < 1 year to 10 years
							Refugee, immigrant, family sponsored, asylum seeker
							Kurdish
67.	Willey 2018 [108]	To explore service provision for Victorian regional refugee families from the perspective of maternal and child health nurses	Australia	Maternal and child primary healthcare	Qualitative descriptive	26 maternal and child health nurses	N/A
						Focus groups, questionnaire	
68.	Wojnar 2015 [109]	To explore the perspective of Somali couples on care and support received during the perinatal period in the United States	United States	Perinatal healthcare	Descriptive phenomenology	26 mothers and 22 fathers	Somalia
						Interviews	LOT: ≤ 5 years
							Refugee
69.	Yelland 2014 [110]	To explore the responsiveness of health services to the social and mental health of Afghan women and men at time of having a baby	Australia	Maternity and early childhood services	Qualitative descriptive	16 mothers and 14 fathers 34 health professionals (midwives, general practitioners, refugee health nurses, maternal and child health nurses, obstetricians, community bicultural workers and other healthcare personnel)	Afghanistan
							LOT: < 1 year to ≥6 years
							Refugee
							Hazera, Tajik, Pashtu, Afghan, Sadath
						Interviews and focus groups	

^a ^For qualitative research in cases where the methodology was not explicitly named or it was unclear, we applied a label based on the description provided. General ‘qualitative’ exploratory studies were categorized as ‘qualitative descriptive’

^b ^The description of migrants is based on what was provided in the paper (country of origin, length of time (LOT*) in country, immigration status, and/or ethnicity); Often ‘immigrant’ is used to generally refer to anyone foreign-born without specifying which immigration statuses are included

Almost all of the papers were published in English, only two were published in French [22, 69] and just over 40% (*n* = 28) were published within the last 5 years (2015–2019). Three papers were discussion papers [57, 76, 99], while the remaining were studies, mostly qualitative research (*n* = 57); five of these were dissertations [52, 80, 82, 95, 105]. Sixteen papers were discussions or studies conducted in the United States, while 13 papers were focused in Australia, followed by 11 in Canada. Papers from Europe reflected a range of countries, including Sweden (5 papers), Finland and the UK (4 papers respectively), and Germany and Ireland (3 papers respectively). (see Table 4).

**Table 4:** Summary of the literature by publication type, location, focus and populations studied

Descriptor	***N*** = 69 papers % (n)
Publication type	
Qualitative research	82.6% (57)
Quantitative research	2.9% (2)
Mixed-methods research	10.1% (7)
Discussion paper	4.3% (3)
Location of study/discussion paper	
Europe^a^	40.6% (28)
United States	23.2% (16)
Australia and New Zealand	20.3% (14)
Canada	15.9% (11)
Focus of service/care/program/intervention examined	
Maternity (prenatal, birth, and postpartum)	69.6% (48)
Early childhood (post-birth up to age five)	23.2% (16)
Maternity and early childhood	7.3% (5)
**Populations studied**	**N = 66 studies % (n)**
Parents	57.6% (38)
Care-providers	19.7% (13)
Care-providers and parents	22.7% (15)
Parents	*N* = 53
Mothers	81.1% (43)
Fathers	1.9% (1)
Both parents^b^	17.0% (9)
Migration source region of parents^c^	N = 53
Sub-Saharan Africa	56.6% (30)
North Africa/Middle East	41.5% (22)
Latin America	32.0% (17)
Caribbean	7.5% (4)
South Asia	43.4% (23)
South East Asia	20.8% (11)
East Asia	17.0% (9)
Eastern Europe/Russia	24.5% (13)
Western Europe/Australia	9.4% (5)
Migration status of parents^c^	N = 53
Immigrant^d^	56.6% (30)
Family	9.4% (5)
Refugee	54.7% (29)
Asylum-seeker	20.8% (11)
Undocumented (non-status migrants)	17.0% (9)

^a^Includes Austria, Belgium, Finland, France, Germany, Greece, Ireland, Italy, Netherlands, Norway, Portugal, Spain, Sweden and the United Kingdom

^b^One study also included grandparents and other members of their community

^c^A study may be counted in more than one category so percentages do not add to 100%

^d^Immigrant was often used as a general term to describe the foreign-born population and so may have included migrants with other statuses (i.e., other than economic immigrants)

The majority of the literature (*n* = 48; 70%) discussed or examined maternity care, services, or interventions (see Table 4). Most of these articles (*n* = 38) generally described experiences in receiving or giving care during pregnancy, childbirth and/or postpartum; 21 of these included only mothers [21, 50, 51, 55, 59, 61, 63, 64, 66, 67, 71, 72, 74, 82, 89, 93, 95–97, 106, 107], one focused only on fathers [85], and another included both parents’ experiences [109]. Eight other articles described the perspectives of healthcare providers, primarily obstetricians, nurses and midwives [53, 58, 65, 77, 78, 84, 87, 104], while six other papers described both migrant mothers’ and healthcare providers’ general experiences [56, 69, 70, 83, 88, 94] (see Table 3 for details).

Eleven papers focused on a specific maternity service or intervention (see Tables 3 and 4). Ten of these were directed towards migrant mothers including doula support [46, 47], a mobile health application to enhance pregnancy well-being for women from the Caribbean (by providing health information and social support) [48, 49], a nurse-practitioner led prenatal program for migrant farm-workers [57], a support group to enhance prenatal and postnatal education [41], a prenatal training course [75], a community-based antenatal service [86], a group pregnancy care initiative for Karen women from Burma [92] and a specialized antenatal clinic for refugee women [100]. One paper examined a culturally tailored prenatal health education video series that was developed for both mothers and fathers from Somalia [68].

Sixteen papers (23% of the literature) focused on care, an intervention or a program during early-childhood (see Tables 3 and 4). These included: studies that examined migrant mothers’ general experiences of accessing primary healthcare [52, 62, 79] or early-childhood programs [105] for their children; one study that explored nurses’ experiences in providing primary healthcare to refugee families with a child aged 0 to 6 years [108]; papers that reported on parents and care-providers’ perspectives of peer support group interventions meant to enhance parenting skills, promote child development and reduce isolation among immigrant mothers [22] and refugee mothers/parents [80, 101–103]; and literature that described and/or evaluated specialized early-education programs for migrant and refugee families, or that investigated the experiences of educators in providing these services [60, 73, 76, 90, 98, 99].

The five remaining papers examined care or programs that spanned the periods of maternity and early-childhood (see Tables 3 and 4). These included: three that examined asylum seeker and refugee mothers’ experiences accessing various health and social services during pregnancy and early motherhood [81, 91, 110]; two of these [91, 110] also considered the perspectives of healthcare professionals, and one also included the experiences of fathers [110]; one article that inquired on mothers’ experiences at a community based perinatal health and social centre which provides medical and psychosocial care to families living in vulnerable contexts from pregnancy up to age five [31]; and another paper that explored the experiences of community workers who provide care and support to refugee and asylum seeker mothers in the perinatal and post-birth periods [54].

The research literature (*n* = 66 studies) primarily reflects parents’ views and experiences with care, services and/or programs/interventions; 80% of studies included parents and 42% included care-providers (see Table 4). Parents’ perspectives are mainly those of mothers’ (*n* = 52 studies); ten studies included fathers [68, 79, 85, 98, 101–103, 105, 109, 110]. The migrant populations studied were mostly from non-western countries; more than half of the studies (57%) included migrant parents from Sub-Saharan Africa. South Asia, North Africa/Middle East and Latin America were represented in 43, 42 and 32% of the research respectively (see Table 4). The most common source countries were Somalia (*n* = 11), Sudan (*n* = 11), Afghanistan (*n* = 10), India (*n* = 9) and Iraq (*n* = 8), and refugees were represented in over half of the studies (55%) (see Table 4).

Regarding the quality of the research, for the qualitative studies, generally it was good- responses to the quality criteria were positive for all of the MMAT items across all papers. The majority of these studies were ‘qualitative descriptive’ (*n* = 37), which was an appropriate approach given most of the studies sought to explore or describe experiences of receiving or providing care. Grounded theory was the methodology used in three studies [67, 69, 78], however ‘theories’ were not generated, raising some question about the validity of using this approach. However, overall the findings across the research were well substantiated by the data and most papers provided in-depth interpretations and discussions of the results.

For the two quantitative studies, one was a small, primarily descriptive, pilot project [68]. The sample of parent participants in this study was relatively small, and based on convenience sampling, but did represent approximately 30% of the target population, which were Somali clients who were receiving care in the obstetrics department where the study was conducted. Details on the number of care-provider participants were not provided. The measurements and analyses were appropriate for the research objectives. The second study, a randomized control trial, only reported preliminary descriptive data about the participants and their participation in the intervention [90]. The methods, nonetheless, were well described, randomization was appropriate, and differences between intervention groups at baseline were explained; one important limitation was that measurement tools were not translated to migrants’ languages which may affect the validity of the eventual outcomes of the research.

For the mixed-methods studies, one study provided inadequate details on the methods and was therefore deemed poor quality [75]; the other six were all good quality- there was a clear rationale for using mixed-methods, and the qualitative and quantitative components were sufficiently described and ‘mixed’ [60, 88, 97, 100–102]. Two strengths across the research, irrespective of the design, were: 1- the inclusion of minority language migrants as participants; of the 53 studies with migrant parents, 36 accommodated those speaking a language other than the host-country language; and 2- the migrant populations were generally well-described using key indicators (i.e., country of origin, length of time, and migration status).

### Transnationalism and care

Across the literature, it was evident that many migrant families value their ‘ways of belonging’ and wish to maintain a transnational identity, especially for their children [21, 31, 49, 67, 69–72, 85, 86, 89, 93–100, 102, 103, 105, 109]. A number of papers also showed that cultural, religious and linguistic ties are often acknowledged and addressed in health and social care. Cultural and religious ways of belonging were attended to in various ways. These included: the use of community-based doulas, mostly other mothers from the same communities, who acted as “cultural bridges” and provided information on different cultural birthing customs [47]; hiring of a cleric to conduct religious traditions and prayers in the hospital [21]; the involvement of peers in support/community groups and programs towards enhancing social support and healthy behaviors through the sharing of knowledge [41, 70, 80, 90–92, 101]; the creation and use of culturally-adapted materials including a prenatal training course [75], a psycho-education program [99], a specialized antenatal clinic [100], perinatal health education videos [68], and a mhealth application developed to support a healthy pregnancy and to connect women to services and other women in their ethnic community [48, 49]; and the adaptation or tailoring of care to respond to different cultural and religious needs and expectations, for example having a female care-provider, asking about food preferences and providing culturally appropriate recommendations on prenatal nutrition, baby care, and parenting [41, 53, 54, 58, 61, 65, 78, 79, 84, 86, 92–95, 97, 98, 103, 106–108, 110].

Language was also addressed in care using different approaches. This was mostly done by accommodating migrants through the use of interpreters, inter-bi-cultural/lingual health workers or liaisons and translated materials [41, 46, 47, 49, 53–55, 58, 61, 65, 73, 76–78, 84, 86, 90–92, 94, 96, 98, 100, 103–108, 110]. Referring women to community supports where care providers speak migrants’ language, was also used to ensure linguistically adapted care [108]. When language could not be accommodated, some care-providers used smiling and having a kind demeanor as a means to make women feel accepted and to show compassion and empathy, regardless of language barriers [59, 104]. Some care-providers made an effort to learn a few words of the migrants’ language to create connection and make women feel more comfortable [53]. Speaking more slowly, adjusting their words and using miming or non-verbal expression was also used when language was a challenge [77–79, 86, 91, 106, 107]. In three papers, care-providers actively encouraged families to speak their native language and to pass it on to their children [31, 101, 105]; in one of these, maintaining culture was also promoted [2]. And lastly, accessing care from care-providers who were from the same ethnic community was also a way for migrants to feel respected and understood in their culturally and linguistically ‘ways of belonging’ [52, 62, 74, 96].

Despite there being evidence that ‘ways of belonging’ are addressed in care, there were also several papers that showed that care-providers are not sufficiently aware and responsive to cultural, religious and linguistic ties. Migrants reported challenges with communication, including not being able to express themselves or to understand information [50, 51, 54, 55, 59, 62, 64, 66, 69, 70, 74, 79, 81, 82, 85, 86, 88, 89, 91, 92, 96, 97, 100, 102, 106, 107, 109, 110], care not being culturally appropriate, for example post-birth and infant care practices not being respected or accommodated, and having unmet expectations due to different experiences in their home countries (e.g., anticipating more or less prenatal visits, ultrasounds and tests) [21, 52, 54–56, 61–64, 66, 67, 69–71, 74, 82, 86, 93, 95–97, 100, 102, 105–107, 110]. Similarly, care-providers also encountered difficulties assessing, understanding and responding to needs due to language and cultural barriers [53, 58, 60, 65, 73, 77, 78, 84, 87, 88, 91, 100, 102, 104, 108]. Across the literature a need for making care more ‘culturally safe’ was identified and deemed essential, including having a more diverse and trained (on cultural competency) healthcare workforce, greater use of linguistic/cultural brokers, and more openness to different ways of doing and/or simply acknowledgement, humility/respect and kindness towards difference [21, 47, 50, 51, 53, 56, 57, 59, 62, 66, 67, 70, 72–74, 77–79, 81, 83, 84, 86–89, 91, 94, 96, 98–102, 104, 106–109].

Other forms of transnational ties (i.e., ‘ways of being’) were mentioned in fewer papers. This included references to families worrying about relatives, including children who remained in the home country [54, 55, 59, 60, 67, 86, 105] and families staying connected via phone/communication technologies and/or visits as a source of social and emotional support, to maintain cultural identity, and/or for assistance with childcare [21, 22, 31, 48, 49, 67, 69, 85, 90, 95, 96, 102, 105]. It also included families sending money back home [105], women seeking healthcare in their country to give birth [21], and families using transnational networks to obtain advice and health information- partly due to local care not being adapted [21, 49, 88, 94, 96, 105].

Overall, there was very little evidence showing that care-providers consider and address ‘ways of being’ in their interventions or care. Some literature showed that care-providers were aware that family separation was causing distress and/or the papers concluded that this issue should be addressed in care [54, 60, 86, 110]. In Yelland (2014), two-thirds of women reported being asked by a doctor, midwife or maternal and child health nurse about their family members in Australia and back home; one participant expressed that she would have liked to have been asked about her parents [110]. In Rickmeyer et al. (2015), care for a refugee woman participating in an early-childhood program included discussing the relationships that a woman maintained with her family back home (a source of support for her). This intervention was comforting for the woman and also enhanced the rapport with the care-provider who was then able to support the woman to work through her trauma [90]. Another paper mentioned that midwives thought that women may be having children in order to receive more governmental family allowance with the intention to use the money to financially support family members back home, although there was no indication on whether this was discussed or addressed with families [77]. In two other papers (by the same authors), the role of family back home as a source of emotional and social support during pregnancy was recognized and incorporated into a mhealth intervention; the application provided a forum for sharing information and could also be used to involve family members in prenatal care visits via conference calls [48, 49].

No studies explicitly examined care-providers’ perceptions of transnational ties, however a number of studies suggest that there is a mix of views, including some healthcare professionals having a positive attitude towards different ways of belonging, including a tolerance and/or openness to different cultures, religions and/or identities [53, 58, 66, 78, 84, 95, 104, 106, 107, 110], and others having the view that migrants should fully assimilate and conform to the healthcare system of the new country, such as learning the country’s language and not having issue with male care-providers [53, 58, 65, 66, 78, 84, 104]. In some instances, healthcare professionals reported feeling left-out when providing care across languages [47, 58]. On the other hand, some migrants felt that their care-providers did not care about them [70] while others reported experiencing stereotyping and discrimination [55, 66, 82, 102, 106, 107, 109]. Care-providers also reported witnessing migrants being treated differently due to their cultural, religious and linguistic differences [87, 88, 104].

Regarding migrants’ transnational ‘ways of being’, it remains unknown what care-providers think- none of the literature revealed how care-providers perceive the connections that migrants maintain with their home country, including social, emotional and economic ties and/or the use of health or other services abroad. However, there is some suggestion that some care-providers are concerned about, and empathize with migrant families that are distressed and worried about their loved ones who have remained in the home country [60]. In this paper, educators working with refugees reported that they found it distressing to hear what families were dealing with and they felt that more support should be provided in this regard. Conversely, there is also a suggestion that some healthcare providers are unsympathetic to migrants’ connections to the home country and/or feel that it is not within their scope of practice to inquire about them [69, 88].

## Discussion

The results of the review show that transnational ties in terms of ‘ways of belonging’ are acknowledged and addressed in care provided to migrant families during pregnancy, postpartum and early childhood, although important gaps remain. There is little evidence however, that transnational ties, in terms of ‘ways of being’, are acknowledged and addressed by care providers. Perceptions of ‘ways of belonging’ appear to be mixed, with some care-providers being open to and willing to adapt care to accommodate religious, cultural and linguistic differences, while others are not. How care-providers perceive the social, emotional and economic ties and/or the use of services abroad, remains relatively unknown.

Significant attention has been given to cultural competency and cultural safety as approaches to improve the quality of care and to reduce health inequities among migrants, as well as indigenous and other ethnic minority groups [111–113]. Cultural competency generally refers to a set of attitudes and practices towards creating health and social care environments where cultural, religious and linguistic ties are respected and accommodated [114]. Cultural safety involves acknowledging and addressing power differentials in clinical interactions and the broader system and structures that result from social, economic, political, and historical circumstances [112]. Results from the current review show that continued efforts are needed to ensure that pregnancy, post-birth and early childhood care and interventions are adapted and culturally safe for migrants (recommendations and some additional resources are reported in Table 5). Given that many migrant families value maintaining a transnational identity, especially for their children, care should also involve supporting migrants to practice their cultural and religious traditions and to speak their native languages. Although there is evidence that diversity is respected and accommodated, it does not appear to be promoted, and emphasis still tends to be on integration (i.e., addressing settlement issues and encouraging the learning of the host-country language and culture) [39–41, 115, 116]. Maintaining a transnational identity, however, is not counter to integration, both processes may occur simultaneously [12], and preserving cultural, religious and linguistic ties may provide an alternate source of belonging, contribute to social and economic capital and have health benefits for migrant families [5, 7, 24, 27, 94]. In turn, these positive outcomes may actually reinforce integration.

**Table 5:** Recommendations for practice and resources

*For supporting ‘Ways of belonging’*	
Increase the diversity of the healthcare workforce	
Offer tailored, community-based programs and services for migrants	
Partner with communities and organizations to develop interventions and programs	
Provide cultural competence training to all health and social care personnel	
Use linguistically and culturally-adapted materials	
Use trained linguistic, cultural brokers	
Reflect on your own culture, beliefs and attitudes about ‘others’	
Recognize and avoid stereotypes	
Attend to power differentials and engage in two-way dialogue	
Humbly acknowledge that you are a learner and remain open to different ways of knowing and doing	
Communicate in a value free, respectful tone	
Tailor care to the context	
Actively counter racism and discrimination	
*For supporting ‘Ways of being’*	
Inquire about family members, including children back home	
Acknowledge transnational stressors (e.g., economic commitments, family responsibilities, stressful relationships)	
Ask about the use of health and social services abroad	
Ask about other health information, support, advice and medicines coming from back home	
*Resources: cultural competency and cultural safety*	
The Office of Minority Health: https://minorityhealth.hhs.gov	
The National Center for Cultural Competence: https://nccc.georgetown.edu	
Health Information Translations, Quality health education resources for diverse populations: https://Healthinfotranslations.org	
EthnoMed: https://ethnomed.org	
Diversity Rx: www.diversityrx.org	
HealthReach, Health information in Many Languages: https://healthreach.nlm.nih.gov	

The review findings are consistent with other research that shows migrant families maintain emotional, social and economic ties with family, and also use health services and receive support from the home country [1]. There is a growing body of research that shows that these transnational ties can have both positive and negative impacts on migrants’ lifestyle behaviours, disease management, social well-being, mental health and information and treatment seeking activities [20, 29, 117–121]. Moreover, migrants have reported having to reconcile differences between advice received from family members back home and care-providers on health related matters in maternal or child care [105]. Evidently, if care-providers do not consider the transnational stresses (concern for family members, economic strain), sources of support and use of healthcare, they will lack a full understanding of migrants’ everyday lives and of the potential impact that these ties may have on migrants’ health. This may then affect care-providers’ relationships with migrant families and/or the effectiveness of their health and social care interventions. Although the review did not shed much light on how exactly these forms of transnational ties should be addressed in care, it did show that inquiring about family members back home could be used to build rapport [90, 110], and that technology could be used to foster the involvement and support of family living far away in maternity care [49]. Without evidence, it is premature to make specific recommendations for care, however, based on the findings, healthcare providers should at minimum, be cognizant of and inquire about migrants’ transnational contexts that may affect their health and well-being (see Table 5).

Care-providers’ views of migrants also have an impact on care. Othering and discrimination towards migrants in the healthcare system based on culture, language, race and religious differences are known issues and affect access to and quality of services [122–124]. These practices involve implicit biases as well as overt racism and discrimination where migrants are viewed differently and provided sub-optimal care, which are justified through explanations of culturalism and racialization [122]. Through culturalism and racialization migrants’ are perceived as inherently inferior due to their worldviews, beliefs and race, and their health and care disparities are seen as being the result of their cultural and religious practices and genetics. In this process migrants are also categorized into differentiated groups and not viewed as individuals, thus further contributing to an ‘us’ vs. ‘them’ dichotomy. Having a ‘foreign’ status, especially being a refugee or not having a legal status, exacerbates this divisiveness and adds to the notion that migrants merit less. Recognizing these biases and harmful ideologies and actively countering racism and discrimination are therefore also key to supporting ways of belonging and creating a care environment that is safe (Table 5). Care-providers can combat racism and discrimination by first being aware of their prejudices and consciously altering their language and behaviours while interacting with migrants [122, 125]. They may also sensitize and educate others by modelling behaviour, speaking openly about issues of racism and discrimination and condemning unjust treatment of migrants [122, 125]. Cultural competency training should also be complemented with anti-racism training.

To further develop care approaches and foster a safe care environment, it would be informative to know what care professionals think about transnational ‘ways of being’- whether they deem economic, social and emotional ties to the home countries as problematic- for example, as a hindrance to integration, or as a factor that contributes to social isolation; and/or whether they view them positively, for example as a source of support and/or for maintaining cultural and linguistic identity. It would also be useful to know whether they consider these to be within their scope of care. While care-providers may be open to accommodating different cultures and languages, they may be less willing to consider and involve people abroad and/or to extend their care to concerns outside their borders.

The review highlighted research gaps. Future research is needed to determine if and how care providers acknowledge and address transnational ‘ways of being’ (social, emotional, economic ties and use of health services) in care during pregnancy, postpartum and early childhood. We also need research to learn about what care-providers think and feel regarding migrants’ ‘ways of being’ and also to hear the perspectives of migrants on how they feel these transnational ties should (or should not) be addressed in care. The viewpoints of both mothers and fathers should be examined since these are likely gendered experiences [119, 121], and since fathers overall are less represented in research on pregnancy, postpartum and early intervention care.

### Limitations and strengths

The searches yielded a vast amount of records to sort through, therefore it is possible that some literature was missed. We only included papers that focused on health promotion generally and with healthy populations, papers that focused on treatments or one aspect of health promotion, and/or ill or disabled populations, may have included pertinent information regarding transnational ties and care. We also did not include grey literature which may have had relevant information as well. It is worthy to note that no papers specifically inquired about transnational ties and care, so the conclusions drawn in this review are based on the examination of study results that generally captured experiences of care and/or descriptions of interventions and programs. Given this indirect approach and the type of data extracted, it was not possible to draw definitive conclusions regarding the extent to which transnational ties are acknowledged and addressed in care or about the views of healthcare professionals on transnational ties. Despite these limitations, we used a thorough approach to identify, select and analyse papers and we synthesized a broad body of literature representing a range of migrant populations living in a number of different countries. We only used primary sources to ensure we could assess data directly, and we included studies that examined parents’ experiences as well as those that examined care-providers’ experiences. As far as we are aware this is the first review to examine transnational ties and health and social care supporting migrant families during pregnancy, postpartum and early childhood.

## Conclusion

To ensure migrants have equitable access to effective, quality care during pregnancy, postpartum and early childhood, further work is needed to raise the critical consciousness of care-providers and to create more culturally safe interventions and care environments. Future research is needed to know whether and how care-providers’ take into account transnational ‘ways of being’, including relationships with children and other family members who remained in the home country, use of health services abroad, and receipt of advice and support from family back home, which may affect their relationships with migrant families and/or the effectiveness of their health and social care interventions. Research is also needed to know the perspectives of care-providers on migrant families’ transnational ‘ways of being’.

## Supplementary information

**Additional file 1.** Database search results.

## Data Availability

Data sharing is not applicable to this article since all data are retrievable from the original sources. Articles included in the review are also summarized in Table 3.

## Abbreviations

ECEC: Early childhood education and care
LOT: Length of time
MCH: Maternal and child health
MMAT: Mixed Methods Appraisal Tool
N/A: Not applicable
