# Supplementary material for: Transnationalism and care of migrant families during pregnancy, postpartum and early-childhood: an integrative review
Source: BMC Health Serv Res. 2020 Aug 24;20:778. doi: 10.1186/s12913-020-05632-5 (PMC7446052; doi:10.1186/s12913-020-05632-5)
Supplement: Supplementary file 1 — Additional file 1. Database search results. [file 12913_2020_5632_MOESM1_ESM.docx]

**Additional File 1: Database search results**

EMBASE: 22 913

Family Studies Abstracts: 310
Global Health: 4 896
MEDLINE: 13 395
PsycINFO: 6201
PubMed: 7 938
Social Sciences Abstracts: 2009
Social Work Abstracts: 208
CINAHL: 6 288
Campbell Collaboration: 0
Dissertations and Thesis: 2 005
Scopus: 14 333
Social services abstracts: 1 211
Sociological abstracts: 2 198
Web of Science: 9 796
